# ﻿Karyotype differentiation and male meiosis in European clades of the spider genus *Pholcus* (Araneae, Pholcidae)

**DOI:** 10.3897/CompCytogen.v16i4.85059

**Published:** 2022-11-02

**Authors:** Jiří Král, Ivalú M. Ávila Herrera, František Šťáhlavský, David Sadílek, Jaroslav Pavelka, Maria Chatzaki, Bernhard A. Huber

**Affiliations:** 1 Laboratory of Arachnid Cytogenetics, Department of Genetics and Microbiology, Faculty of Science, Charles University, Viničná 5, 128 44 Prague 2, Czech Republic Charles University Prague Czech Republic; 2 Department of Zoology, Faculty of Science, Charles University, Viničná 7, 128 44 Prague 2, Czech Republic Charles University Prague 2 Czech Republic; 3 Centre of Biology, Geosciences and Environmental Education, University of West Bohemia, Univerzitní 8, 306 14 Plzeň, Czech Republic University of West Bohemia Plzeň Czech Republic; 4 Department of Molecular Biology and Genetics, Democritus University of Thrace, 68100 Alexandroupolis, Greece Democritus University of Thrace Alexandroupolis Greece; 5 Alexander Koenig Zoological Research Museum, Adenauerallee 127, 53113 Bonn, Germany Alexander Koenig Zoological Research Museum Bonn Germany

**Keywords:** haplogyne, inversion, NOR, rDNA, sex chromosome, speciation, Synspermiata

## Abstract

Haplogyne araneomorphs are a diverse spider clade. Their karyotypes are usually predominated by biarmed (i.e., metacentric and submetacentric) chromosomes and have a specific sex chromosome system, X_1_X_2_Y. These features are probably ancestral for haplogynes. Nucleolus organizer regions (NORs) spread frequently from autosomes to sex chromosomes in these spiders. This study focuses on pholcids (Pholcidae), a highly diverse haplogyne family. Despite considerable recent progress in pholcid cytogenetics, knowledge on many clades remains insufficient including the most species-rich pholcid genus, *Pholcus* Walckenaer, 1805. To characterize the karyotype differentiation of *Pholcus* in Europe, we compared karyotypes, sex chromosomes, NORs, and male meiosis of seven species [*P.alticeps* Spassky, 1932; *P.creticus* Senglet, 1971; *P.dentatus* Wunderlich, 1995; *P.fuerteventurensis* Wunderlich, 1992; *P.phalangioides* (Fuesslin, 1775); *P.opilionoides* (Schrank, 1781); *P.silvai* Wunderlich, 1995] representing the dominant species groups in this region. The species studied show several features ancestral for *Pholcus*, namely the 2n♂ = 25, the X_1_X_2_Y system, and a karyotype predominated by biarmed chromosomes. Most taxa have a large acrocentric NOR-bearing pair, which evolved from a biarmed pair by a pericentric inversion. In some lineages, the acrocentric pair reverted to biarmed. Closely related species often differ in the morphology of some chromosome pairs, probably resulting from pericentric inversions and/or translocations. Such rearrangements have been implicated in the formation of reproductive barriers. While the X_1_ and Y chromosomes retain their ancestral metacentric morphology, the X_2_ chromosome shows a derived (acrocentric or subtelocentric) morphology. Pairing of this element is usually modified during male meiosis. NOR patterns are very diverse. The ancestral karyotype of *Pholcus* contained five or six terminal NORs including three X chromosome-linked loci. The number of NORs has been frequently reduced during evolution. In the Macaronesian clade, there is only a single NOR-bearing pair. Sex chromosome-linked NORs are lost in Madeiran species and in *P.creticus*. Our study revealed two cytotypes in the synanthropic species *P.phalangioides* (Madeiran and Czech), which differ by their NOR pattern and chromosome morphology. In the Czech cytotype, the large acrocentric pair was transformed into a biarmed pair by pericentric inversion.

## ﻿Introduction

Spiders exhibit an enormous species diversity, paralleled by high karyotype diversity. However, despite considerable recent progress (e.g., Král et al. 2006, 2013, 2019; Araujo et al. 2012; Kořínková and Král 2013; Ávila Herrera et al. 2021), our knowledge of spider cytogenetics is still fragmentary. Most data on spider chromosomes concern entelegyne araneomorphs, which include the large majority of the described spider species. The cytogenetics of the other clades (mesotheles, mygalomorphs, haplogyne araneomorphs) is much less understood (Kořínková and Král 2013; Ávila Herrera et al. 2021).

Haplogyne araneomorphs (“haplogynes”) consist of the Synspermiata clade and two families, Filistatidae and Hypochilidae (Wheeler et al. 2017; Shao and Li 2018). Haplogynes currently include more than 6150 described species placed in 20 families (based on data of World Spider Catalog 2022). Haplogynes exhibit a considerable karyotype diversity. Their diploid numbers range from 2n♂ = 5 (*Afrilobus* sp., Orsolobidae) to 2n♂ = 152 (*Caponianatalensis* O. Pickard-Cambridge, 1874, Caponiidae), which are the lowest and highest diploid numbers in spiders, respectively (Král et al. 2019). Their karyotypes are composed of monocentric (i.e., standard) chromosomes except for the superfamily Dysderoidea whose chromosomes are holokinetic (holocentric) (Král et al. 2019). Holokinetic chromosomes lack a localized centromere (Mola and Papeschi 2006). Karyotypes of haplogynes with monocentric chromosomes are usually predominated by biarmed (i.e., metacentric and submetacentric) chromosomes (Král et al. 2006; Ávila Herrera et al. 2021). Furthermore, the prophase of the male first meiotic division includes the so-called diffuse stage (Kořínková and Král 2013), characterized by a considerable decondensation of autosomes and overcondensation of sex chromosomes (Benavente and Wettstein 1980; Král et al. 2006; Ávila Herrera et al. 2021). Haplogynes exhibit a variety of sex chromosome systems. Male sex chromosomes include one or several elements that do not recombine during meiosis and are presumably nonhomologous. The peculiar X_1_X_2_Y system has been found in seven families (Král et al. 2006, 2019; Ávila Herrera et al. 2016, 2021; Paula-Neto et al. 2017; Araujo et al. 2020). It is probably ancestral for araneomorph spiders including haplogynes (Paula-Neto et al. 2017; Ávila Herrera et al. 2021). The ancestral structure of the X_1_X_2_Y system probably comprises two large metacentric X chromosomes and a metacentric Y microchromosome, which display a specific achiasmatic end-to-end pairing during male meiosis (Ávila Herrera et al. 2021). The origin of the X_1_X_2_Y system is unresolved. In some clades, it has converted into other sex chromosome systems (Král et al. 2006, 2019; Ávila Herrera et al. 2016, 2021). Besides non-recombining elements, spider sex chromosomes probably also contain a chromosome pair formed by the chromosomes X and Y, which recombine and show a very low level of differentiation (cryptic sex chromosome pair, CSCP) (Kořínková and Král 2013). Haplogynes also vary greatly in the number and location of nucleolus organizer regions (NORs) (Král et al. 2006; Ávila Herrera et al. 2021). These structures contain genes for 18S, 5.8S and 28S rRNA (Sumner 2003). The number of NORs ranges from one to nine; their position is usually terminal; and they spread frequently from autosomes to sex chromosomes (Král et al. 2006; Ávila Herrera et al. 2021).

The present study focuses on the cytogenetics of pholcid spiders (Pholcidae), the most diversified haplogyne family with monocentric chromosomes. This family currently comprises almost 1900 described species in 97 genera (World Spider Catalog 2022). Pholcids occur on all continents except Antarctica. Most species inhabit tropical and subtropical regions; some species are synanthropic (Huber 2011). From a cytogenetic point of view, pholcids are the best-explored group of haplogynes. A total of 64 species have been karyotyped, including 11 species determined to genus level only (based on The Spider Cytogenetic Database 2022). Despite this, our knowledge on karyotype evolution remains insufficient for many pholcid clades, including the most species-rich genus, *Pholcus* Walckenaer, 1805 (with currently more than 350 species; World Spider Catalog 2022). To reduce this gap, we studied the differentiation of karyotype, sex chromosomes, and NORs as well as the course of male meiosis in the dominant species groups of *Pholcus* present in mainland Europe, Crete, and Macaronesia. Nucleolus organizer regions have previously been studied in few spider species. More comprehensive data on the evolution of these structures are only available from pholcids (Ávila Herrera et al. 2021).

We paid specific attention to the Macaronesian clade of *Pholcus*. Macaronesia consists of five volcanic archipelagos in the Atlantic Ocean, west of the Iberian Peninsula and northwestern Africa. *Pholcus* is among the most species-rich genera of Macaronesian spiders. The Macaronesian clade currently includes more than 20 described species that are largely restricted to the Canaries and Madeira (Dimitrov and Ribera 2007; Dimitrov et al. 2008; Huber 2011). This clade exhibits an enormous diversification rate, among the highest found in spiders (Dimitrov et al. 2008).

Our aim is to determine the fundamental traits of karyotype evolution in European clades of *Pholcus*. Based on our new findings and on previously published data, we explore the congruence of individual karyotype markers with published phylogenies and discuss the possible evolutionary implications of karyotype transformations.

## ﻿Material and methods

### ﻿Spider specimens

Information on the studied species (number of analyzed specimens, their sex, and locality data) is given in Table 1. Voucher specimens are deposited in the Zoological Research Museum Alexander Koenig, Bonn (Germany).

**Table 1.** Species studied, with specimen number, sex, and geographic origin. Abbreviation: sad = subadult.

Taxon	Individuals	Locality	GPS Coordinates (Latitude, Longitude)
*P.crypticolens*/*opilionoides* species group			
*P.creticus*	4♂	Greece, Crete, Topolia, Topolia cave	35.4119, 23.6817
	2♂	Greece, Crete, Stavros, Lera cave	35.5908, 24.1023
*P.opilionoides*	4♂	Czech Republic, Veselí nad Lužnicí	49.1506, 14.6930
*P.phalangioides* species group			
*P.alticeps*	8♂	Czech Republic, Chomutov	50.4527, 13.4166
*P.phalangioides*	1♂	Portugal, Madeira, Santana	32.8043, -16.8855
Macaronesian species group			
*P.fuerteventurensis*	2♂	Spain, Canariens, Fuerteventura, Giniginamar	28.2024, -14.0734
*P.dentatus*	1 sad ♂, 1♂	Portugal, Madeira, Achadas da Cruz	32.8390, -17.1907
*P.silvai*	2♂	Portugal, Madeira, Levada das 25 fontes	32.7611, -17.1374

### ﻿Preparation of chromosomes, determination of karyotype

Chromosome preparations were obtained from testes of adult males by a modification of the spreading technique described by Dolejš et al. (2011). The gonads were dissected and immersed into a hypotonic solution (0.075M KCl) for 20–25 min at room temperature (RT). Hypotonization was followed by two fixations in ethanol:acetic acid (3:1) for 10 and 20 min (RT), respectively. Subsequently, tissue was placed in a drop of 60% acetic acid on a clean slide and quickly shredded with a pair of tungsten needles to obtain a cell suspension. Finally, the slide was placed on a warm (40 °C) histological plate. The drop of dispersed tissue evaporated while being moved constantly by a tungsten needle. Slides were stained using 5% Giemsa solution in Sörensen buffer (pH 6.8) for 28 min (RT). They were studied under an Olympus BX 50 microscope equipped with DP 71 CCD camera (Olympus, Tokyo, Japan). To construct the karyotype, the morphology of metaphase II chromosomes was analyzed. Sister metaphases II (5 plates) were evaluated using the IMAGE TOOL 3.0 software (https://imagetool.software.informer.com/3.0/). Relative chromosome length was estimated as a percentage of the total chromosome length of the haploid set (TCL). This set also included sex chromosomes X_1_, X_2_, and Y. Karyotypes were assembled using the COREL PHOTO PAINT X3 programme. Determination of the sex chromosome system was based on data from male meiosis (segregation of sex chromosomes and their behavior in prophase and metaphase I). The X_2_ and Y chromosomes were similar in size. Therefore, we used a paired samples Wilcoxon test to analyse their size difference. It was impossible to distinguish the CSCP from autosomes by light microscopy. Therefore, the CSCP and autosomes are referred to collectively as chromosome pairs.

### ﻿Detection of nucleolus organizer regions (NORs)

The NOR pattern was determined by fluorescent in situ hybridisation (FISH) with a 18S rDNA probe from *Dysderaerythrina* (Walckenaer, 1802) (Dysderidae) (see Ávila Herrera et al. 2021 for details of probe). Whereas the previously common method of NOR-detection by silver staining only visualizes NOR sites transcribed during the preceding interphase (Miller et al. 1976), NOR detection by a rDNA probe gives more accurate results. The probe was generated following Sadílek et al. (2015). The 18S rRNA gene fragment was amplified by polymerase chain reaction (PCR) from genomic DNA using forward and reverse primers 5´-CGAGCGCTTTTATTAGACCA-3´ and 5´-GGTTCACCTACGGAAACCTT-3´, respectively. The PCR product was extracted using the Wizard SV Gel and PCR Clean-Up System (Promega), re-amplified by PCR, and labeled with biotin-14-dUTP by nick translation using a Nick Translation Kit (Abbott Molecular).

FISH was performed with the biotinylated 18S rDNA probe as described by Fuková et al. (2005). Chromosome preparations were pre-treated with 100 μg/ml RNase A in 2× saline-sodium citrate (SSC) buffer (1 h, 37 °C). Chromosomes were denatured (3 min 30 s, 68 °C) by 70% formamide in 2×SSC. The probe mixture contained 20 ng of 18S rDNA and 25 μg of salmon sperm DNA (Sigma-Aldrich, Burlington, MA, USA) in 5 μl of 50% formamide and 5 μl of 20% dextran sulphate per slide. Biotin labelled 18S rDNA was detected with Cy3-streptavidin (Jackson ImmunoRes. Labs Inc., West Grove, PA, USA), with signal amplification by biotinylated antistreptavidin and Cy3-streptavidin (Vector Labs Inc., Burlingame, CA, USA). The preparations were counterstained with Fluoroshield containing 4´,6-diamidino-2-phenylindole (DAPI) (Sigma-Aldrich, Burlington, MA, USA). Considering the sensitivity of pholcid chromosomes to denaturation, two procedures were used to reduce this process. First, the slides were placed in an incubator for 1 hour (60 °C) before the experiment. Second, denaturation time was reduced (3 min). Preparations were observed under the Olympus IX81 microscope (Olympus, Tokyo, Japan) equipped with an ORCA-AG monochromatic camera (Hamamatsu, Hamamatsu, Japan). The images were pseudocolored (red for Cy3 and light green for DAPI) with Cell^R software (Olympus Soft Imaging Solutions GmbH, Münster, Germany).

## ﻿Results

### ﻿Karyotype data

The male karyotype of all species studied had 25 predominantly metacentric chromosomes and the X_1_X_2_Y system (2n♂ = 25, X_1_X_2_Y). The X_1_ was the longest element of the set. Chromosomes X_2_ and Y were medium-sized elements of similar size. Chromosome pairs decreased gradually in length (Suppl. material 1).

#### *Pholcuscrypticolens*/*opilionoides* species group

**Figure 1.**
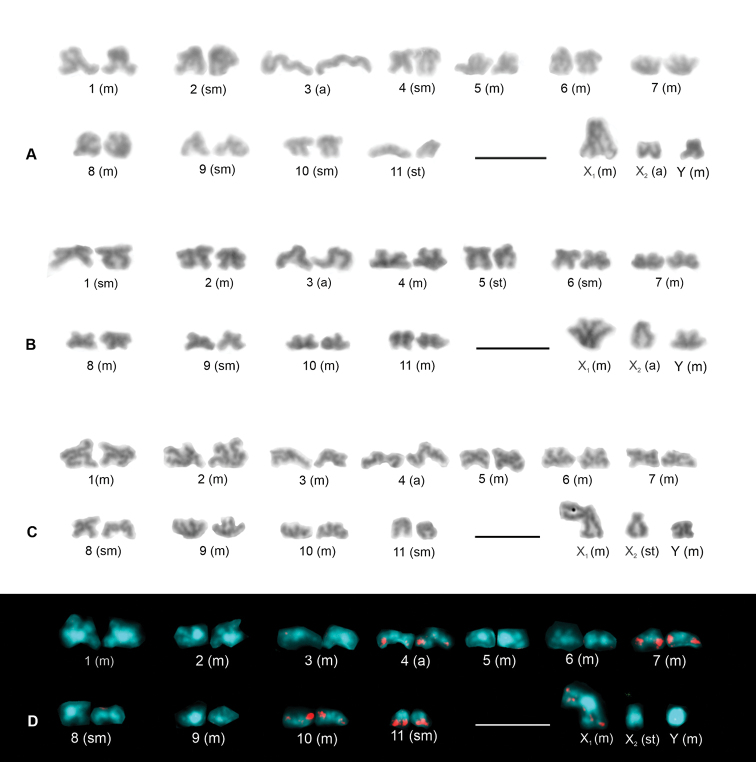
*Pholcuscrypticolens*/*opilionoides* and *phalangioides* groups, male karyotypes (**A–C** stained by Giemsa **D**FISH). Based on sister metaphases II **A***P.creticus***B***P.alticeps***C, D***P.phalangioides* (Madeira) **C** standard karyotype **D** karyotype, detection of NORs. Prepared from the same plate as the standard karyotype. Note four chromosome pairs with terminal NOR (nos 4,7,10,11) and the X_1_ chromosome with NOR at both ends. Pairs nos. 7, 10, and 11 are biarmed, pair no. 4 is acrocentric. NORs are localized at the long arm of these pairs. Scale bars: 10 μm.

**Figure 2.**
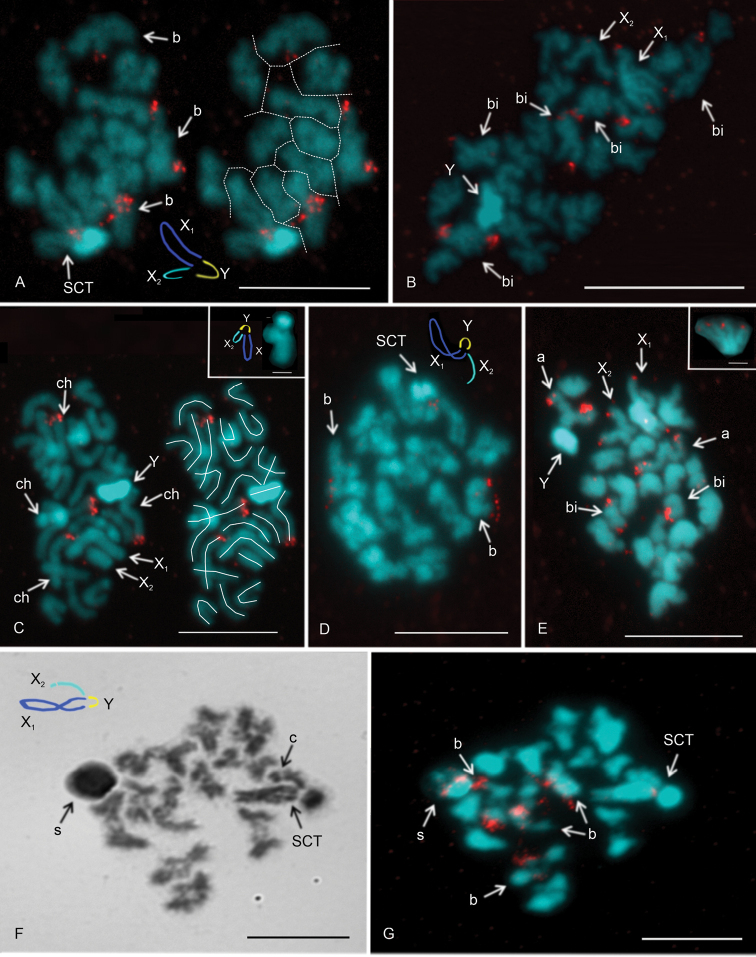
*Pholcuscrypticolens*/*opilionoides* and *phalangioides* groups, males, NOR detection **A–E, G**FISH**F** Giemsa staining **A, B***P.opilionoides***A** diplotene. Three bivalents contain NOR. There is also a signal on the sex chromosome trivalent. Y chromosome overcondensed. Note the scheme of sex chromosome pairing and scheme of the plate (particular elements separated by a dotted line) **B** two fused sister metaphases II. Note the terminal signal on five biarmed elements belonging to chromosome pairs. Odd number of chromosomes with signal suggests that NOR locus of one chromosome pair is heterozygous for NOR cluster. The X_1_ chromosome includes NOR at both ends **C***P.creticus*, mitotic metaphase. Two chromosome pairs contain a terminal NOR. Y chromosome overcondensed. On the right side: scheme of the plate (particular chromosomes marked by a line). Inset: metaphase I, sex chromosome trivalent (without signal). Note the scheme of sex chromosome pairing **D, E***P.alticeps***D** metaphase I. Two bivalents contain NOR. There is also signal on the sex chromosome trivalent. Y chromosome overcondensed. Note the scheme of sex chromosome pairing **E** two fused sister metaphases II, Y chromosome overcondensed. NOR bearing elements: one pair of biarmed chromosomes (a terminal NOR), one pair of acrocentric chromosomes (a terminal NOR at long arm), X_2_ chromosome (a terminal NOR at long arm), X_1_ chromosome (NOR at both ends). Inset: X_1_ chromosome (from another plate), note the NOR at both ends **F, G***P.phalangioides*, Madeira, metaphase I. Four bivalents include a NOR. There is also a signal on the sex chromosome trivalent. Note the scheme of sex chromosome trivalent. Abbreviations: a = chromosome of the acrocentric pair bearing NOR, b = bivalent containing NOR, bi = chromosome of a biarmed pair bearing NOR, c = centromere, ch = chromosome bearing NOR, s = sperm nucleus, SCT = sex chromosome trivalent, X_1_ = X_1_ chromosome, X_2_ = X_2_ chromosome, Y = Y chromosome. Scale bars: 10 μm except for insets (5 μm).

The chromosome pairs of the males of *P.creticus* comprised five metacentric (nos 1, 5–8), four submetacentric (nos 2,4,9,10), one subtelocentric (no. 11), and one acrocentric pair (no. 3). Sex chromosomes were metacentric except for the acrocentric X_2_ (Fig. 1A). Lenghts of the X_2_ and Y chromosomes differed significantly (paired samples Wilcoxon test, W = 0, P < 0.001). The Y chromosome was longer than the X_2_ (Suppl. material 1). This species had two chromosome pairs with a terminal NOR each (Fig. 2C). The morphology of these pairs is unresolved.

The chromosomes of the males of *P.opilionoides* exhibited the same morphology as in populations studied previously (Ávila Herrera et al. 2021). They were metacentric except for five submetacentric chromosome pairs (nos 2–6) and an acrocentric X_2_ chromosome. The lengths of the X_2_ and Y chromosomes differed significantly (paired samples Wilcoxon test, W = 0, P < 0.001). The Y was shorter than the X_2_. We succeeded in determining the NOR pattern in one specimen. The karyotype contained three biarmed chromosome pairs bearing a terminal NOR each. One pair was heterozygous for a NOR cluster. Furthermore, a small NOR was also detected at each end of the X_1_ chromosome (Fig. 2A, B).

#### *Pholcusphalangioides* species group

The male karyotype of *P.alticeps* consisted of metacentric chromosomes except for three submetacentric (nos 1,6,9), one subtelocentric (no. 5), and one acrocentric (no. 3) chromosome pairs as well as the acrocentric X_2_ chromosome (Fig. 1B). The lengths of the X_2_ and Y chromosomes did not differ significantly (paired samples Wilcoxon test, W = 1, 0.10 < P < 0.20). The karyotype included two chromosome pairs with a terminal NOR locus each. While one NOR-bearing pair was formed by small biarmed chromosomes, the other one consisted of large acrocentric chromosomes with a NOR at the end of the long arm. The karyotype contained three terminal sex chromosome-linked NORs (two on the X_1_ chromosome and one at the end of the long arm of the X_2_ chromosome) (Fig. 2D, E).

The karyotype of the single male of *P.phalangioides* from Madeira consisted of metacentric chromosomes except for two submetacentric (nos 8 and 11) and one acrocentric pair (no. 4) as well as a subtelocentric X_2_ (Fig. 1C). The lengths of the X_2_ and Y chromosomes did not differ significantly (paired samples Wilcoxon test, W = 2, 0.10 < P < 0.20). Three biarmed (nos 7,10,11) and one acrocentric chromosome pairs (no. 4) contained a terminal NOR each, which was placed at the end of the long arm. Beside this, a NOR was also found at each end of the X_1_ chromosome (Figs 1D, 2F, G).

#### Macaronesian species group

**Figure 3.**
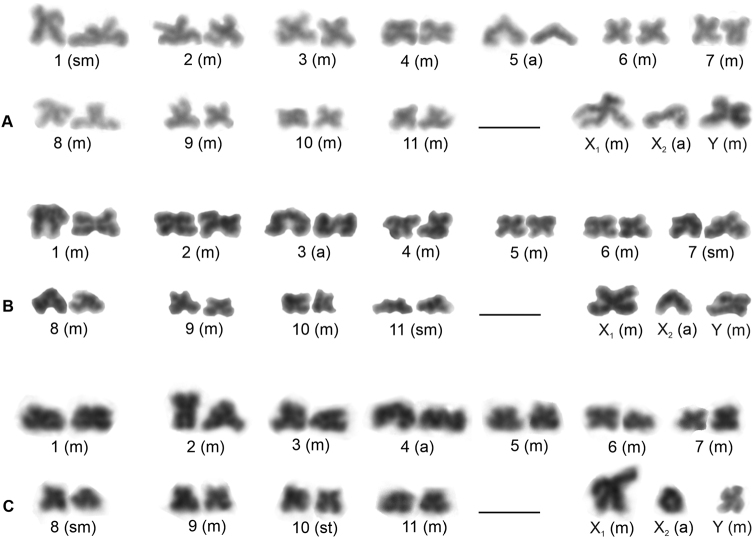
*Pholcus*, Macaronesian group, male karyotypes, stained by Giemsa. Based on sister metaphases II **A***P.fuerteventurensis***B***P.dentatus***C***P.silvai*. Scale bars: 10 μm.

**Figure 4.**
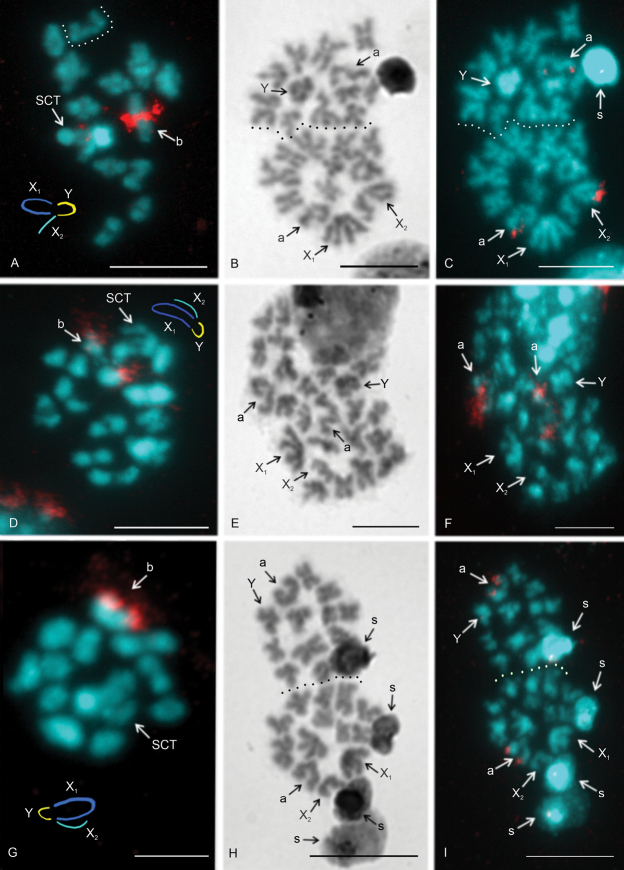
*Pholcus*, Macaronesian group, NOR detection **A, C, D, F, G, I**FISH**B, E, H** Giemsa staining **A–C***P.fuerteventurensis***A** metaphase I (a bivalent belonging to another plate is separated by a dotted line). One bivalent contains a NOR. There is also a signal on the sex chromosome trivalent. Note the scheme of sex chromosome pairing **B, C** two sister metaphases II separated by a dotted line. Note two terminal NORs, one on the long arm of the acrocentric pair and another one on the long arm of the acrocentric X_2_ chromosome **D–F***P.dentatus***D** metaphase I, one large bivalent contains a terminal NOR. Note the scheme of sex chromosome pairing **E, F** two fused metaphases II. Long arm of the acrocentric pair contains terminal NOR. Sister chromatids of chromosomes of this pair are sometimes associated by NOR clusters (see the right chromosome of the pair) **G–I***P.silvai***G** metaphase I, one bivalent involves a terminal NOR. Note the scheme of sex chromosome pairing **H, I** two metaphases II separated by dotted line. Long arm of the acrocentric pair contains terminal NOR. Abbreviations: a = chromosome of the acrocentric pair bearing NOR, b = bivalent containing NOR, s = sperm nucleus, SCT = sex chromosome trivalent, X_1_ = X_1_ chromosome, X_2_ = X_2_ chromosome, Y = Y chromosome. Scale bars: 10 μm.

The karyotype of *P.fuerteventurensis* from the Canaries was composed of metacentric chromosomes except for one submetacentric (no. 1) and one acrocentric pair (no. 5) as well as an acrocentric X_2_ chromosome (Fig. 3A). The lengths of the X_2_ and Y chromosomes did not differ significantly (paired samples Wilcoxon test, W = 5, P > 0.2). *P.fuerteventurensis* had a single large acrocentric NOR-bearing pair containing a NOR at the end of the long arm. A NOR was also placed at the end of the long arm of the X_2_ chromosome (Fig. 4A–C).

In *P.dentatus* from Madeira, the chromosome pairs were metacentric except for two submetacentric (nos 7 and 11) and one acrocentric pair (no. 3). The sex chromosomes had a metacentric morphology except for the acrocentric X_2_ (Fig. 3B). The lengths of the X_2_ and Y chromosomes differed significantly (paired samples Wilcoxon test, W = 0, P < 0.001). The X_2_ was longer than the Y (Suppl. material 1).

The chromosome complement of the second Madeiran species, *P.silvai*, had metacentric chromosomes except for one submetacentric (no. 8), one subtelocentric (no. 10), one acrocentric pair (no. 4), and an acrocentric X_2_ chromosome (Fig. 3C). The lengths of the X_2_ and Y chromosomes differed significantly (paired samples Wilcoxon test, W = 0, P < 0.001). The Y was larger than the X_2_ chromosome (Suppl. material 1).

Both Madeiran species showed the same NOR pattern, namely a single locus at the end of the long arm of the acrocentric pair (Fig. 4D–I).

### ﻿Sex chromosome behavior in male germline

**Figure 5.**
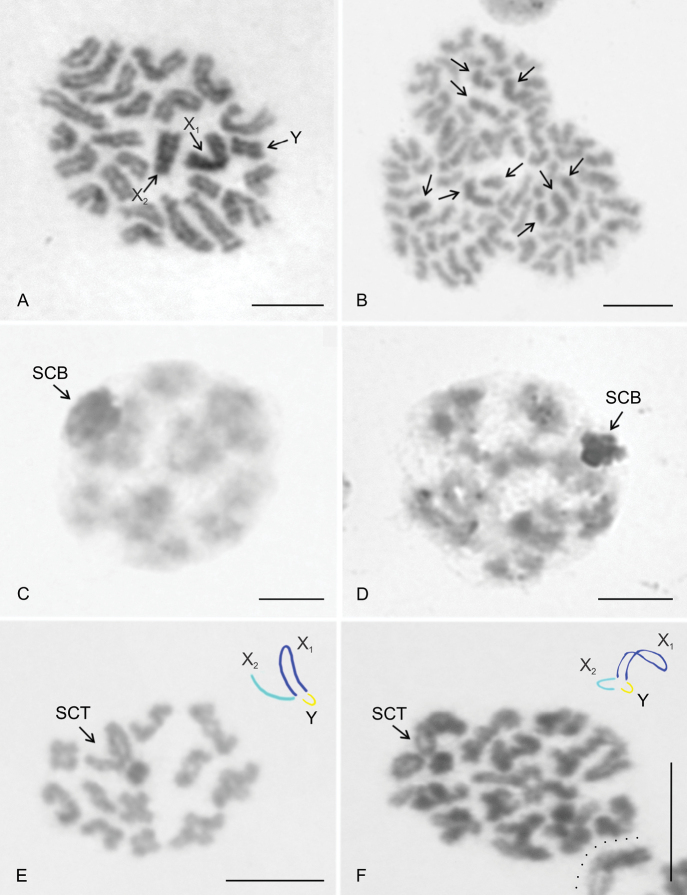
*Pholcus*, males, sex chromosome behavior at spermatogonial mitosis and first meiotic division, Giemsa staining **A***P.dentatus*, spermatogonial metaphase. Note the association of positively heteropycnotic sex chromosomes in the middle of the plate **B***P.silvai*, early spermatogonial anaphase, three half plates. Sex chromosomes exhibit a slight positive heteropycnosis and are placed in the middle of the half plates. Sex chromosomes are marked by arrows **C***P.fuerteventurensis*, early diffuse stage. Sex chromosomes form a positively heteropycnotic body on the periphery of the nucleus **D***P.silvai*, late diffuse stage. The sex chromosome body on the periphery of the nucleus exhibits positive heteropycnosis **E***P.fuerteventurensis*, diakinesis (11 bivalents and a X_1_X_2_Y trivalent). The Y chromosome stained more intensively than the X chromosomes. Note the scheme of sex chromosome pairing **F***P.alticeps*, diplotene (11 bivalents and a X_1_X_2_Y trivalent). Edge of another diplotene separated by dotted line. Note the scheme of sex chromosome pairing. Abbreviations: SCB = sex chromosome body, SCT = sex chromosome trivalent, X_1_ = X_1_ chromosome, X_2_ = X_2_ chromosome, Y = Y chromosome. Scale bars: 10 μm.

**Figure 6.**
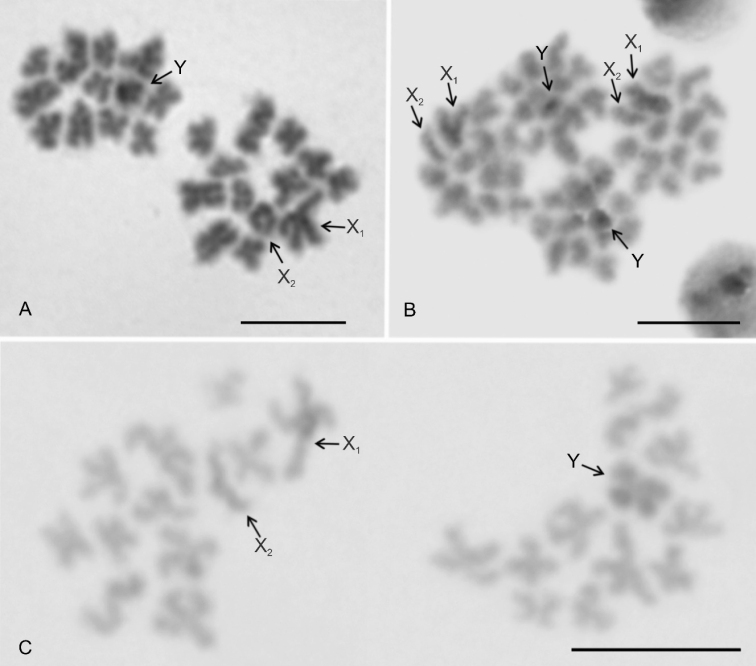
*Pholcus*, males, sex chromosome behavior in second meiotic division, Giemsa staining **A***P.silvai*, two sister metaphases II. Metaphase II containing the X chromosomes is composed of 13 chromosomes. Metaphase II containing the Y chromosome comprises 12 chromosomes **B***P.alticeps*, two sister anaphases II. Chromosomes X_1_ and Y display positive heteropycnosis. The X chromosomes are associated. The Y chromosome is placed in the middle of the half plates **C***P.fuerteventurensis*, two sister metaphases II. Plate containing the X chromosomes is incomplete (1 chromosome missing). Note the positive heteropycnosis of the sex chromosomes. Abbreviations: X_1_ = X_1_ chromosome, X_2_ = X_2_ chromosome, Y = Y chromosome. Scale bars: 10 μm.

In general, the behavior of the sex chromosomes was characterized by positive heteropycnosis (i.e., more intensive staining) and association (i.e. close proximity of chromosomes without pairing) which transformed into pairing in some phases. The specific behavior of sex chromosomes was initiated as early as in spermatogonial mitosis. Sex chromosomes often exhibited positive heteropycnosis and a loose association in spermatogonial prophases, metaphases, and anaphases (Fig. 5A, B). During metaphase (Fig. 5A) as well as on anaphase half-plates (Fig. 5B), they were often placed in the middle of the plates. They remained overcondensed and positively heteropycnotic during premeiotic interphase, early prophase I (leptotene-pachytene), and diffuse stage. During this period, they often formed a body on the periphery of the plate (Fig. 5C, D). Bivalents were fuzzy and spherical during the early diffuse stage (Fig. 5C). However, towards the end of the diffuse stage, they showed chiasmata and their morphology was similar to that found during late prophase I (Fig. 5D). During late prophase I (diplotene-diakinesis) and metaphase I, the condensation of the sex chromosomes decreased. The Y chromosome was often more condensed than the X chromosomes and bivalents (Fig. 5E). The pattern of heteropycnosis also varied during metaphase II. While in the Madeiran species the sex chromosomes usually exhibited none or only indistinct heteropycnosis (Fig. 6A), they were often positively heteropycnotic in *P.fuerteventurensis* from the Canaries and in species from mainland Europe (Fig. 6C). The Y chromosome often showed a more intensive staining than the X chromosomes. All species were characterized by sex chromosome heteropycnosis during anaphase II whereas heteropycnosis of the X_2_ chromosome was indistinct in some plates (Fig. 6B).

In the premeiotic interphase, the association of sex chromosomes transformed into sex chromosome pairing. The mode of sex chromosome pairing was most apparent during late prophase and metaphase I. Both ends of the metacentric sex chromosomes, X_1_ and Y, took part in pairing (Fig. 5E, F). The pairing pattern of the monoarmed X_2_ chromosome differed among species. In *P.creticus* (and in some plates of *P.alticeps* and *P.dentatus*), both ends of the X_2_ chromosome were involved in pairing (Fig. 5F). The same pattern of pairing was found in *P.opilionoides* during early diplotene (Fig. 2A). After that, pairing was restricted to the long arm of the X_2_ chromosome. In other species, only the long arm of the X_2_ chromosome was involved in pairing, by its end (Fig. 5E); this pattern was also observed in the absence of hypotonization. The X chromosomes were usually arranged in parallel during anaphase I, metaphase II, and anaphase II (Fig. 6B). The Y chromosome was placed in the middle of the half-plates during anaphase II (Fig. 6B).

## ﻿Discussion

Pholcids are the most diversified family of haplogyne spiders with monocentric chromosomes and a suitable model group to study karyotype evolution. Their distribution is worldwide, and the available molecular phylogeny is the most comprehensive among all major spider families (Eberle et al. 2018). They are currently the best-explored family of haplogynes from a cytogenetic point of view. Closely related species often differ in their karyotypes, suggesting the involvement of chromosome rearrangements in the formation of interspecific barriers (Ávila Herrera et al. 2021).

Here we focus on karyotype differentiation of the genus *Pholcus*. Previously published cytogenetic data concern seven species determined to species level and two species determined to genus level only (The Spider Cytogenetic Database 2022). With five newly studied species, our study increases the number of cytogenetically analyzed *Pholcus* species to 14. However, karyotype data of three species are in all probability incorrect (Table 2). These data are analysed in detail by Ávila Herrera et al. (2021). The karyotyped representatives determined to species level represent five of the clades proposed for the genus (Huber et al. 2018), namely the *P.bicornutus*, *P.crypticolens*/*P.opilionoides*, *P.guineensis*, *P.phalangioides*, and Macaronesian groups.

**Table 2.** Summary of *Pholcus* cytogenetic data. Doubtful data in bold. In most of these cases, it is possible to deduce probable correct information (in parentheses). †see Ávila Herrera et al. (2021: 22) for discussion of sex chromosome system. ‡See Ávila Herrera et al. (2021) for discussion of sex chromosome system (p. 23) and morphology of chromosome pairs (p. 21). §See Ávila Herrera et al. (2021) for discussion of number of chromosome pairs (p. 18) and sex chromosome system (p. 22). Abbreviations: a = acrocentric, bi = biarmed, CP = chromosome pair, m = metacentric, p = short chromosome arm, q = long chromosome arm, SC = sex chromosome, SCS = sex chromosome system, sm = submetacentric, st = subtelocentric, t = terminal, ? = unknown.

Taxon	2n	SCS	Chromosome pairs: number, morphology	Sex chromosome morphology	NOR number (CP/SC)	NOR-bearing CPs: number, morphology (NOR location)	NOR-bearing sex chromosomes: morphology (NOR location)	References
*bicornutus* species group								
*P.pagbilao*	23	X_1_X_2_Y	7m+3sm	X_1_m+X_2_a+Ysm	5/0	3 bi (t);1 bi (1 NOR p, t + 1 NOR q, t)		Ávila Herrera et al. 2021
*crypticolens*/*opilionoides* species group								
*P.creticus*	25	X_1_X_2_Y	5m+4sm+1st+1a	X_1_m+X_2_a+Ym	2/0	2 (t)		this study
*P.crypticolens*†	**24**	**X_1_X_2_0**	most or all m	X_1_?+X_2_?				Suzuki 1954
	(25)	(X_1_X_2_Y)						
*P.manueli*‡	25	**X0** (X_1_X_2_Y)	**11a**	**Xsm**				Wang et al. 1997
*P.opilionoides*	25	X_1_X_2_Y	6m+5sm	X_1_m+X_2_a+Ym	3/2	3 bi (t)	X_1_ m (1 NOR p, t + 1 NOR q, t)	Ávila Herrera et al. 2021, this study
*guineensis* species group (+ *P.bamboutos*)								
*P.bamboutos*	23	X_1_X_2_Y	most bi	X_1_m+X_2_m+Ym				Ávila Herrera et al. 2021
*P.kindia*	23	X_1_X_2_Y	8m+1sm+1st	X_1_m+X_2_m+Ym				Ávila Herrera et al. 2021
Macaronesian species group								
*P.dentatus*	25	X_1_X_2_Y	8m+2sm+1a	X_1_m+X_2_a+Ym	1/0	1a (q, t)		this study
*P.fuerteventurensis*	25	X_1_X_2_Y	9m+1sm+1a	X_1_m+X_2_a+Ym	1/1	1a (q, t)	X_2_ a (1 NOR q, t)	this study
*P.silvai*	25	X_1_X_2_Y	8m+1sm+1st+1a	X_1_m+X_2_a+Ym	1/0	1a (q, t)		this study
*phalangioides* species group								
*P.alticeps*	25	X_1_X_2_Y	6m+3sm+1st+1a	X_1_m+X_2_a+Ym	2/3	1 bi (t); 1a (q, t)	X_1_ m (1 NOR p, t + 1 NOR q, t);	this study
							X_2_ a (1 NOR q, t)	
*P.phalangioides* (Czech cytotype)	25	X_1_X_2_Y	9m+2sm	X_1_m+X_2_sm+Ym	3/3	3 bi (t)	X_1_ m (1 NOR p, t + 1 NOR q, t);	Král et al. 2006, Ávila Herrera et al. 2021
							X_2_ sm (q, t)	
*P.phalangioides* (Madeiran cytotype)	25	X_1_X_2_Y	8m+2sm+1a	X_1_m+X_2_st+Ym	4/2	3 bi (q, t); 1 a (q, t)	X_1_ m (1 NOR p, t + 1 NOR q, t)	this study
species determined to the genus level only								
*Pholcus* sp. (India)§	**26**(?)	**X_1_X_2_0** (X_1_X_2_Y)						Sharma and Parida 1987
*Pholcus* sp. (Kazakhstan)	25	X_1_X_2_Y	7m+3sm+1a	X_1_m+X_2_st+Ym				Ávila Herrera et al. 2021

### ﻿Diploid numbers and morphology of chromosome pairs

The ancestral pholcid karyotype probably consisted of 15 chromosome pairs and the sex chromosomes X_1_, X_2_, and Y (Ávila Herrera et al. 2021). Like many other spider groups (Suzuki 1954; Král et al. 2006, 2013), some pholcid clades show a trend towards a decrease in chromosome number (Ávila Herrera et al. 2021). This is also probably how the ancestral karyotype of the subfamily Pholcinae has evolved with its 11 chromosome pairs and sex chromosomes X_1_, X_2_, and Y. This karyotype is also ancestral for *Pholcus* (Ávila Herrera et al. 2021). It was found in all karyotyped clades of the genus except for the *P.bicornutus* and *P.guineensis* groups (Ávila Herrera et al. 2021; this study). In the latter two species groups, the number of chromosome pairs decreased further to ten. This feature could be a synapomorphy of a large group within *Pholcus* comprising the Subsaharan African, Southeast Asian, and Australasian groups of this genus (Ávila Herrera et al. 2021).

The chromosome pairs of ancestral pholcids probably had a biarmed morphology (Ávila Herrera et al. 2021). Most pairs were probably metacentric. Chromosome pairs of *Pholcus* species are predominated by biarmed chromosomes except for *P.manueli* Gertsch, 1937 (Wang et al. 1997). However, the information on this species is based only on the pattern of constitutive heterochromatin. Therefore, it should be reanalyzed by determination of chromosome morphology at the mitotic metaphase or metaphase II (Ávila Herrera et al. 2021).

The karyotype of the unidentified *Pholcus* sp. from Kazakhstan reported in Ávila Herrera et al. (2021) contains a large acrocentric pair, which was supposed to be an apomorphy of this species. Kazakhstan is inhabited by representatives of the *P.crypticolens*/*opilionoides* and *P.ponticus* groups (Huber 2011). Our study revealed that the acrocentric pair is in fact more common in Eurasian *Pholcus* groups with the karyotype formula 25, X_1_X_2_Y. The pair is the third, fourth or fifth by size and its relative length ranges from 7.20 to 8.22% of TCL (Ávila Herrera et al. 2021; this study). The end of the long arm of this pair contains a NOR (see discussion on NOR evolution below). The large acrocentric pair has most probably originated by a pericentric inversion from a biarmed one. In the present study, it was found in representatives of all analyzed groups. This pattern suggests that the large acrocentric pair could be a synapomorphy of several species groups within the genus with the karyotype formula 25, X_1_X_2_Y. A further interesting pattern was found in *P.phalangioides*. While the cytotype from Madeira retained the large acrocentric pair, in the Czech cytotype this pair had reverted to biarmed, thus the karyotype was again composed exclusively of biarmed chromosomes. Since the chromosome pairs of the above mentioned cytotypes differed only by this reversion, it most probably resulted from a pericentric inversion. Furthermore, the reversion of an acrocentric pair to biarmed had also occurred in *P.opilionoides* whose karyotype is also formed exclusively by biarmed chromosomes. The acrocentric pair is not present in karyotypes of the *Pholcus* lineages with the formula 23, X_1_X_2_Y. However, a reversion of an acrocentric pair to non-acrocentric cannot be ruled out in ancestors of these lineages. If such a scenario is correct, the large acrocentric pair would be a synapomorphy of the entire genus *Pholcus*. This marker has not been found in the sister clade of *Pholcus*, i.e. the *Micropholcus*/*Leptopholcus* clade (Ávila Herrera et al. 2021). However, the large acrocentric pair could even have been present in the ancestral karyotype of the *Micropholcus*/*Leptopholcus* clade. The karyotypes of this clade have been derived from the supposed ancestral karyotype of pholcines (25, X_1_X_2_Y) by multiple fusions of chromosome pairs. The large acrocentric pair could have been involved into these fusions.

Closely related species of *Pholcus* often differ by the morphology of one or several chromosome pairs. For example, *P.fuerteventurensis* from the Canaries (belonging to the Macaronesian clade) differs from species of the same clade from Madeira by the morphology of three pairs. A possible apomorphy of *P.fuerteventurensis* is the transformation of the largest chromosome pair from metacentric to submetacentric. The Madeiran species show two possible synapomorphies, namely transformations of two metacentric pairs into submetacentric or subtelocentric. The first transformation concerned the 7^th^ pair of *P.dentatus* and the 8^th^ pair of *P.silvai*, respectively. The second transformation concerned the 11^th^ pair of *P.dentatus* and the 10^th^ pair of *P.silvai*, respectively (Suppl. material 1). Even greater are the differences found between *P.opilionoides* and *P.creticus* from the *P.crypticolens*/*opilionoides* clade. A possible synapomorphy of these species is the change of two metacentric pairs to submetacentric (2^nd^ and 4^th^ pairs). While the large acrocentric pair has been retained in *P.creticus*, it has converted to biarmed in *P.opilionoides*. Moreover, both species differ by the morphology of five other chromosome pairs (Suppl. material 1). Potential synapomorphies of *P.alticeps*, *P.phalangioides* (*P.phalangioides* group) and *Pholcus* sp. from Kazakhstan include changes of two metacentric pairs into submetacentric. The first change concerned probably the 6^th^ pair of *P.alticeps*, the 8^th^ pair of *P.phalangioides* (the 7^th^ pair in Ávila Herrera et al. 2021), and the 7^th^ pair of *Pholcus* sp. The second change concerned probably the 9^th^ pair of *P.alticeps*, the 11^th^ pair of *P.phalangioides* (the 10^th^ pair in Ávila Herrera et al. 2021), and the 9^th^ pair of *Pholcus* sp.

A similar karyotype differentiation, where the morphology of one or more chromosome pairs changed while the number of chromosome pairs remained the same, has also been found in other pholcid genera (Ávila Herrera et al. 2021). These changes in morphology occurred most probably by pericentric inversions or translocations. These rearrangements leave the chromosome number unchanged and they can often result in reproductive isolation (Rieseberg 2001; Ayala et al. 2005).

### ﻿Sex chromosomes

All *Pholcus* species studied so far exhibit the X_1_X_2_Y system (Král et al. 2006; Ávila Herrera et al. 2021, this study). Many haplogynes with the X_1_X_2_Y system have retained its ancestral type with two large metacentric X chromosomes and a metacentric microchromosome Y (Ávila Herrera et al. 2021).

The genus *Pholcus*, like most other pholcids with the X_1_X_2_Y system (Ávila Herrera et al. 2021), is conservative in having a metacentric X_1_ chromosome, which is the largest chromosome of the set. In *Pholcus* species with the karyotype 25, X_1_X_2_Y, the size of the X_1_ ranges from 9.87 to 14.37% of TCL (Ávila Herrera et al. 2021; this study). The size of the Y chromosome has increased considerably in a clade of the subfamily Pholcinae including *Quamtana* Huber, 2003, *Muruta* Huber, 2018, *Leptopholcus* Simon, 1893, and *Pholcus*. In general, the Y chromosome can increase in size by accumulation of constitutive heterochromatin, rearrangements between autosomes and sex chromosomes, or by a combination of these events (e.g., Kejnovský et al. 2009; Schartl et al. 2016). Available data suggest a major role of heterochromatin accumulation in the expansion of the pholcine Y chromosome. The Y chromosome of *P.phalangioides* is composed exclusively of constitutive heterochromatin (Král et al. 2006). A reinterpretation of karyotype data obtained by Wang et al. (1997) suggests the same composition of the Y chromosome in *P.manueli* (Ávila Herrera et al. 2021). Constitutive heterochromatin is a very dynamic part of the genome. The size of heterochromatic blocks could change even within populations (Sumner 1990). Although the Y chromosome of *Pholcus* is formed exclusively by heterochromatin, its size is relatively stable in this genus ranging from 4.77 to 7.10% of TCL except for *P.kindia* Huber, 2011 (11.72% of TCL) (Ávila Herrera et al. 2021; this study). Particular *Pholcus* species might differ by the extent of condensation in the Y chromosome, which contributes to its diversity in size. The enormous increase in size of the Y chromosome in *P.kindia* was probably caused by insertions of autosomal material (Ávila Herrera et al. 2021). Among other spiders with the X_1_X_2_Y system, a considerable increase of the Y chromosome size has only been found in one representative of pacullid spiders (Král et al. 2019).

The increase of Y chromosome size in pholcines has been accompanied by a reduction of the X_2_ chromosome. The X_2_ and Y chromosomes exhibit a similar size in the *Pholcus* clades analyzed in this study. The X_2_ chromosome is the most dynamic chromosome of the X_1_X_2_Y system in pholcids. It exhibits a considerable diversity in size and morphology (Ávila Herrera et al. 2021). The ancestral metacentric morphology of the X_2_ chromosome has changed frequently to submetacentric or even monoarmed, probably by pericentric inversions or translocations (Ávila Herrera et al. 2021). As already mentioned, these rearrangements can play a role in the formation of reproductive barriers. This effect is even stronger if the rearrangement concerns sex chromosomes (Presgraves 2008; Kitano et al. 2009; Hooper et al. 2019). The ancestral X_2_ chromosome of *Pholcus* was probably metacentric as found in *P.guineensis* and *P.bamboutos* Huber, 2011 (23, X_1_X_2_Y). This hypothesis is supported by the biarmed morphology of the X_2_ chromosome in the closest relatives of *Pholcus* (Ávila Herrera et al. 2021). During following evolution, the morphology of the X_2_ chromosome gradually changed to acrocentric. This scenario is supported by the non-acrocentric morphology of this element in two species with the formula 25, X_1_X_2_Y, *P.phalangioides* (submetacentric or subtelocentric X_2_) and *Pholcus* sp. (subtelocentric X_2_). The size of the X_2_ chromosome ranges from 5.53 to 6.56% of TCL in species with this formula (Ávila Herrera et al. 2021; this study).

Interestingly, Madeiran and central European specimens of *P.phalangioides* differed slightly in the morphology of the X_2_ chromosome. While the X_2_ chromosome of the Czech *P.phalangioides* was submetacentric (centromeric index 2.85), the X_2_ of the Madeiran specimen was subtelocentric (centromeric index 3.96) (Ávila Herrera et al. 2021; this study). This change in morphology might result from chromosome rearrangement or addition of heterochromatin. The acrocentric morphology of the X_2_ chromosome observed in some metaphases II of *P.phalangioides* is an artifact resulting from precocious separation of chromatids of this chromosome.

The sex chromosomes in *Pholcus* show a specific behavior in the male germline, which, like in other pholcids, includes positive heteropycnosis (more intensive staining), preferential location, and association or pairing. The association and heteropycnosis of sex chromosomes occur as early as during spermatogonial mitosis. Moreover, the sex chromosomes are usually located in the middle of spermatogonial plates, specifically on the metaphase plates (Král et al. 2006; Ávila Herrera et al. 2021; this study) and anaphase half plates (this study). Such behavior in spermatogonial anaphase has not been reported so far and it might occur in other spider species as well, not only in the taxa with the X_1_X_2_Y system. Due to its short duration, the spermatogonial anaphase is only rarely observed, which precludes analysis of sex chromosome behavior during this period. During the premiotic interphase in pholcids, the sex chromosome association evolves into pairing that continues up to metaphase I (Král et al. 2006; Ávila Herrera et al. 2021; this study). Chromosomes of the X_1_X_2_Y system are usually located at the periphery of the plate during early prophase I and diffuse stage. In contrast to that, during late prophase I and metaphase I, they tend to be in the middle of the plate. After segregation of the X and Y chromosomes, the X chromosomes are associated till the end of meiosis. The Y chromosome is usually located in the middle of half plates during anaphase II. Sex chromosomes are positively heteropycnotic only in some phases of meiosis (Ávila Herrera et al. 2021; this study).

Metacentric chromosomes of the X_1_X_2_Y system pair without chiasmata in male meiosis, namely by the ends of both arms (Silva et al. 2002; Král et al. 2006; Ávila Herrera et al. 2021). In some species with a non-metacentric X_2_ chromosome, both chromosome ends remain involved in chromosome pairing. In other species, however, the non-metacentric X_2_ chromosome only pairs by the end of its long arm (Král et al. 2006; Ávila Herrera et al. 2021; this study). In *P.creticus*, both ends of the acrocentric X_2_ chromosome take part in pairing. In *P.alticeps* and *P.dentatus*, which share the morphology of the X_2_ chromosome with *P.creticus*, pairing by both ends was only observed in a small proportion of the cells probably because the pairing of the shorter arm is less stable and loosens during the hypotonization and fixation step of chromosome preparation. In *P.opilionoides*, pairing of the X_2_ chromosome by both ends was only observed in the early diplotene; afterwards, the chromosome paired only by its long arm. In other *Pholcus* species with a monoarmed X_2_ chromosome, only the long arm of X_2_ was involved in pairing (Ávila Herrera et al. 2021; this study). This pattern was observed even in the absence of hypotonization (this study), which indicates that it is not an artifact.

### ﻿NORs

So far, NORs have only been detected in a low number of spider species (see Forman et al. 2013; Král et al. 2013 for references), especially by the means of FISH (see Šťáhlavský et al. 2020; Reyes Lerma et al. 2021 for references). In pholcids, however, NOR patterns have been determined recently in many species by FISH (Ávila Herrera et al. 2021), which makes it possible to contextualize our data with previous knowledge on the NOR evolution in this family. Pholcid spiders show a highly variable numbers of NORs (one to nine), which in the majority of pholcids occur on chromosome ends (Ávila Herrera et al. 2021). Their terminal position suggests that the NORs spread within the karyotype mostly by ectopic recombination, which is most effective in telomeric areas (Goldman and Lichten 1996). NOR bearing pairs in pholcids have a biarmed morphology except for the acrocentric pair found in the present study in most *Pholcus* species with the karyotype formula 25, X_1_X_2_Y. Unlike in other spiders, the spreading of NORs to sex chromosomes is quite common in haplogynes (including pholcids, where it has occurred at least five times) (Král et al. 2006; Ávila Herrera et al. 2021).

The ancestral pattern of the subfamily Pholcinae probably involves three chromosome pairs with a terminal NOR each. Prior to the separation of *Aetana* Huber, 2005, a NOR locus appeared on one end of the X_1_. Thereafter, the NORs gradually spread to the other end of the X_1_ chromosome and to the end of the long arm of the X_2_, i.e., to the regions that ensure the achiasmatic pairing of the sex chromosomes. We assume that the sex chromosome-linked NORs (SCL-NORs) take part in this pairing (Ávila Herrera et al. 2021), probably together with the sequences of the Y chromosome invading the end of the X_2_ (Sember et al. 2020).

Our study reveals a considerable diversity of NOR patterns in *Pholcus*. Based on data from *Pholcus* and the closely related genera, we suppose that the ancestral NOR pattern of *Pholcus* probably comprised two or three chromosome pairs with a terminal NOR locus each and three terminal X chromosome-linked loci (two on the X_1_ chromosome and one on the X_2_). The number of loci has then increased in some species and decreased in others (Ávila Herrera et al. 2021; this study). In *P.pagbilao* Huber, 2011, four NOR bearing pairs have been found, one of them with two terminal NORs (Ávila Herrera et al. 2021). Four NOR-bearing pairs were also found in the Madeiran cytotype of *P.phalangioides* (this study).

A reduction in the number of NORs has occurred repeatedly in *Pholcus*, both on chromosome pairs and on chromosomes of the X_1_X_2_Y system. Thus, the Macaronesian clade exhibits a single acrocentric NOR-bearing pair. *P.fuerteventurensis* from the Canaries retained a single SCL-NOR located at the end of the X_2_ chromosome. The two Madeiran species share a degeneration/loss of SCL-NORs. In the *P.crypticolens*/*opilionoides* group, the reduction was more extensive in SCL-NORs than in NORs located on chromosome pairs. The pattern of *P.opilionoides* differs from the supposed ancestral pattern only by the absence of the X_2_-linked NOR, while the pattern of *P.creticus* is more derived, the SCL-NORs are degenerated/lost (this study). In *P.pagbilao* (*P.bicornutus* group), the number of NOR-bearing chromosome pairs has increased to four whereas SCL-NORs were degenerated/lost (Ávila Herrera et al. 2021). Remarkably, particular clades differ in their pattern of reduction of SCL-NORs. In the *P.phalangioides* and *P.crypticolens*/*opilionoides* groups, the X_2_-linked NOR has been degenerated/lost first. In the Macaronesian clade, however the degeneration/loss has first affected the X_1_-linked NORs (this study). The rDNA sequences responsible for achiasmatic pairing of sex chromosomes could be retained even after degeneration of SCL-NORs, as already reported from the males of *Drosophila* Fallén, 1823 (Roy et al. 2005). The reasons for the repeated degeneration of SCL-NORs in *Pholcus* are unclear. All species without SCL-NORs are island species. Island populations are frequently reduced and thus experience genetic drift, which could lead to random fixation of sex chromosomes without NORs. Moreover, genetic drift is more effective in case of sex chromosomes whose number in the population is reduced in comparison with autosomes (Johnson and Lachance 2012). Within the subfamily Pholcinae, the loss of the SCL-NORs had also occurred in a clade including *Canticus* and *Micropholcus*. In this case, the loss of these NORs has been accompanied by a conversion of the X_1_X_2_Y system to X0 (Ávila Herrera et al. 2021).

### ﻿Karyotype diversity in *P.phalangioides*

*P.phalangioides* showed intraspecific diversity of the NOR pattern and chromosome morphology. Considering NORs, the Czech cytotype exhibited the supposedly ancestral pattern of *Pholcus* (Ávila Herrera et al. 2021). In the Madeiran cytotype, the number of the NOR-bearing pairs has increased to four, each pair containing one NOR locus. The NOR on the X_2_ chromosome has been lost. Intraspecific variability in the NOR number has not previously been reported from pholcids, but it could be expected based on the occurrence of heterozygotes for number of NORs in some species (Ávila Herrera et al. 2021).

The karyotype differences between the Czech and Madeiran cytotype were, however, more profound. They also differed in the morphology of some chromosomes. The chromosome pairs of the Madeiran cytotype showed the original pattern; they included a large acrocentric pair, which has changed to biarmed in the Czech cytotype. Furthermore, both cytotypes differed to some extent in the morphology of the X_2_ chromosome. Intraspecific differences in chromosome morphology have not been previously reported from pholcids. Whether the presence of different cytotypes is in any way related to the apparent *COI* polymorphism in this species (documented in the sequences deposited at NCBI) is unknown. The status of both cytotypes should be further analysed using larger samples and approaches of integrative taxonomy.

## ﻿Conclusions

We present new data on karyotypes and meiotic division of seven species of the genus *Pholcus* (Pholcidae) from Europe. The selected species represent several different species groups within the region whose relationships among each other remain largely unknown. The male karyotype is composed of 25 chromosomes with a X_1_X_2_Y sex chromosome system. The sex chromosomes pair without chiasmata during male meiosis. The karyotypes are predominated by biarmed chromosomes. The karyotypes of most species contain an acrocentric chromosome pair, which has changed to biarmed in some taxa. This marker is either a synapomorphy of the species groups included in this study or a synapomorphy of the genus *Pholcus*. Closely related species usually differ in the morphology of one or several chromosome pairs, which suggests the operation of pericentric inversions and/or translocations. Such rearrangements have been implicated in speciation. The chromosomes X_1_ and Y show a metacentric morphology. By contrast, the X_2_ chromosome is usually acrocentric. NOR patterns are very diversified. In the ancestor of *Pholcus*, these structures were located both on chromosome pairs and on sex chromosomes. Sex chromosome-linked NORs could be involved in the pairing of sex chromosomes. Most of the analyzed species show a specific pattern of NORs. Nucleolus organizer regions have often been degenerated/lost during evolution. Remarkably, the loss seems to preferably affect SCL-NORs. The reason for this phenomenon is unclear. The rDNA sequences crucial for sex chromosome pairing might remain unaffected by the degeneration. *P.phalangioides* yielded two cytotypes, which differ in their chromosome morphology and NOR pattern. Some of the detected chromosome changes appear phylogenetically informative. Although the Macaronesian clade shows a very high rate of speciation, species of this lineage do not differ substantially in the number of chromosome changes from other analyzed lineages of *Pholcus*. However, this conclusion needs to be corroborated by an analysis of more species and species groups.

## Supplementary materials

Supplementary material 1 Species studied, male karyotype data (including standard deviation) Data type Table (MS Excel file) Explanation note Abbreviations: parameters = parameters used to describe chromosome morphology [CI = centromeric index, RCL = relative chromosome length (% of TCL)], specimens = number of specimens used to obtain data (*specimens from Stavros were analysed). Chromosome morphology is indicated by background colour of a box (pink: metacentric, brown: submetacentric, dark blue: subtelocentric, light blue: acrocentric). https://binary.pensoft.net/file/764877This dataset is made available under the Open Database License (http://opendatacommons.org/licenses/odbl/1.0/). The Open Database License (ODbL) is a license agreement intended to allow users to freely share, modify, and use this Dataset while maintaining this same freedom for others, provided that the original source and author(s) are credited.Jiří Král, Ivalú M. Ávila Herrera, František Šťáhlavský, David Sadílek, Jaroslav Pavelka, Maria Chatzaki, Bernhard A. Huber
